# Dosimetric Outcomes of Definitive Radiation Therapy to Anal Squamous Cell Carcinoma in a Single Institution Using Volumetric Modulated Arc Therapy Evaluated Against Australian and International Treatment Planning Guidelines

**DOI:** 10.1002/jmrs.70124

**Published:** 2026-10-29

**Authors:** Faisal Alshamrani, Michael Hsieh, Anthony Karl, Lauren Chamberlain, Stuart Greenham, Josie Goodworth, Maree Wood, Thomas Shakespeare, Tue Le, Abdul Mohd Tahir

**Affiliations:** ^1^ Department of Radiation Oncology, Mid North Coast Cancer Institute Coffs Harbour Health Campus Coffs Harbour New South Wales Australia; ^2^ RCS Faculty of Medicine University of New South Wales Coffs Harbour New South Wales Australia

**Keywords:** anal cancer, anal squamous cell carcinoma, radiation oncology, radiation therapy, volumetric modulated arc therapy

## Abstract

**Introduction:**

Volumetric Modulated Arc Therapy (VMAT) is the technique of choice for anal Squamous Cell Carcinoma (SCC), offering highly conformal dosimetry while improving dose sparing to Organs at Risk (OARs). Achieving the dose constraints listed in national and international guidelines can be challenging. This report validates the achievability of these constraints and brings a clinical context to these guidelines.

**Methods:**

A retrospective analysis of 43 clinician approved plans was conducted. These plans were evaluated against the Radiation Therapy Oncology Workgroup (RTOG) 0529, eviQ, the Assessment of New Radiation Oncology Treatments and Technologies (ANROTAT) and departmental protocol. A constraint was considered ‘pass’ if it met an ideal objective or minor violation. A constraint was considered ‘failed’ if a major violation occurred.

**Results:**

RTOG 0529 Planning Target Volumes (PTV) constraints D98 ≥ 80% and D95 ≥ 90% of the Total Dose (TD) were achieved in 100% of our patient cohort, with a median of 93.8% and 96% TD for the high‐risk PTV (54Gy) and 92.6% and 95.2% TD for the low‐risk PTV (45Gy). The stricter ANROTAT D98 ≥ 90% TD was achieved in 93% of our patient cohort for the high‐risk PTV (54Gy) and 77% for the low‐risk PTV (45Gy). Major violations of the gastrointestinal (GI) OAR constraints were observed in up to 40% of the plans. While there were no strict major violation specifications for skeletal and genitourinary (GU) OARs, dose constraint non‐compliance occurred in up to 19% and 56% of the plans respectively.

**Conclusions:**

Although VMAT is the gold standard for anal SCC, achieving the recommended dose constraints was challenging. Further refinement of dose constraints is essential to ensure the clinical achievability of planning objectives.

## Graphical abstract

This study evaluated 43 clinically approved VMAT plans for anal squamous cell carcinoma (SCC) against RTOG, eviQ, ANROTAT and departmental planning guidelines to assess the achievability of current dose constraints. While most target coverage and median OAR dose objectives were within protocol, not all constraints were consistently met, supporting the proposal of clinically informed and modified dose objectives.

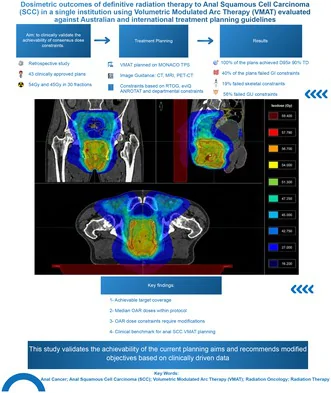

## 1 Introduction

Intensity Modulated Radiation Therapy (IMRT) using fixed‐field beams has been shown to reduce dose to organs at risk (OAR) and improve target volume coverage in radiation treatment for anal cancers compared to three‐dimensional conformal radiotherapy (3DCRT), thus reducing acute and late toxicities [1, 2]. Fixed‐field IMRT planning has since been compared in silico to Volumetric Modulated Arc Therapy (VMAT) in several studies, demonstrating reduced doses to OAR with VMAT [3, 4, 5]. Several Australian and international guidelines have made recommendations towards planning aims, target volume coverage and OAR constraints [6, 7]. The Assessment of New Radiation Oncology Treatments and Technologies (ANROTAT) study analysed dosimetric outcomes with fixed field IMRT across multiple institutions [8]. The Radiation Therapy Oncology Workgroup (RTOG) 0529 also provided target volume and OAR guidelines [1, 2]. However, limited reports to date have evaluated the achievability of the planning aims recommended by these guidelines utilising VMAT, with respect to dosimetric outcomes. The present study validates the achievability of planning aims recommended by RTOG 0529, ANROTAT and eviQ against our departmental protocol using clinician approved treatment plans in a single institution.

## 2 Methods

Following institutional ethics approval (NCNSW 2021/ETH00346) all radiation oncologist (RO)‐approved treatment plans of patients treated with VMAT technique for definitive radiation therapy for anal SCC from 2013 to 2023 were reviewed. Baseline patient and tumour characteristics were obtained through review of electronic medical records (MOSAIQ, Elekta AB, Stockholm, Sweden). Dosimetric parameters including target volume coverage and doses to OARs were obtained from the treatment planning system (Monaco, V6.1.4, Elekta AB, Stockholm, Sweden) and compared against RTOG 0529, eviQ, ANROTAT and our departmental protocol. Our medical physicists and radiation therapists (RT) collaborated to develop a script which utilises the Application Programing Interface function on Monaco V6.1.4. This allowed automated collection of dosimetric data for each target volume and OAR for each individual RO‐approved treatment plan. Plans were scored as having ‘failed’ a planning aim if a major violation was encountered. Minor violations were considered as ‘pass’. Supporting Information of the planning aims are presented in detail in Appendix S1. Patient survival outcomes and toxicities were reported in a separate study [9].

### 2.1 Treatment Planning

All patients were planned uniformly on the same Mid North Coast Cancer Institute (MNCCI) departmental planning protocol, where the planning aims were based upon eviQ guidelines, RTOG 0529 and ANROTAT [1, 8, 10]. Pre‐simulation, all patients were discussed at multidisciplinary (MDT) meetings, and staged using baseline Magnetic Resonance Imaging (MRI) of the pelvis and Fludeoxyglucose F18 Positron Emission Tomography (FDG PET). For Computed Tomography (CT) simulation, patients were positioned head in, supine, hands on chest, legs straight and immobilised with an indexed headrest and foot stocks. Self‐bolusing of the gluteal and perineal region provided adequate build‐up. Patients were scanned with a moderately full bladder with the volume recorded by use of a bladder scanner, and an empty rectum with a diameter < 5 cm assessed on the topograms and mini‐CT scans prior to acquiring the planning CT scan. If the rectum was > 5 cm in diameter, our protocol mandated several steps to reduce rectal size prior to re‐simulating, including voluntary re‐emptying, regular macrogol and simeticone for 2–7 days prior to rescanning or an enema. Pelvis CT was acquired with a scan level of L1/L2 to mid‐thigh using 2 mm slices with intravenous (IV) and oral contrast. Diagnostic MRI and PET were fused to the planning CT to aid target volume delineation. A single radiation oncologist (RO) contoured all target volumes according to the Australasian Gastrointestinal Trials Group (AGITG) guidelines [6]. OARs, including small and large bowel loops, femoral heads, iliac crests, external genitalia and bladder, were contoured by RTs according to the AGITG guidelines and checked by the treating RO. All patients received simultaneous integrated boost (SIB) to two dose levels: 54Gy in 30 fractions to the high‐risk planning target volume (HR‐PTV) and 45Gy in 30 fractions to the low‐risk (LR‐PTV). A uniform 7–10 mm isotropic expansion upon each clinical target volume (CTV) was applied to create the respective PTV. To spare non‐target skin, PTVs were trimmed to 3–5 mm within the skin surface except the skin in the perineum region.

VMAT was employed utilising the Monaco treatment planning system (Monaco, V3 to 6.1.4, Monte Carlo, Elekta AB, Stockholm, Sweden). In general, the isocentre was placed at the centre of the LR‐PTV to ensure adequate image acquisition of the PTVs in all planes. Each plan was optimised using two to three 360‐degree arcs, with a 45‐degree collimator angle. The minimum segment width was set to 1 cm. Medium fluence smoothing was selected. Statistical uncertainty was set to 1% per calculation. All plans were planned using 6MV with a calculation grid of 3 × 3 × 3 mm. Each plan was optimised by an RT and peer checked by an independent experienced RT prior to presenting to the RO for plan approval. Once a plan was assessed and approved by the RO, the plan was data exported by the planning RT then checked by a separate independent experienced RT for final plan approval. Each plan with major violations would undergo initial review by the planning senior before being presented to the treating RO, ensuring optimal plan refinement. Our institution ensures target coverage takes precedence over meeting OAR dose constraints in the treatment of anal SCC, which is consistent with RTOG 0529, ANROTAT and eviQ. In instances of major violations, a discussion would occur with the treating RO, who prioritised maintaining sufficient PTV coverage while minimising radiation exposure to OARs.

In line with the RTOG and ANROTAT guidelines, the OAR priority from highest to lowest was as follows: small bowel, femoral heads, iliac crests, external genitalia, bladder and large bowel. Appendix S2 provides an example of an approved VMAT plan. Quality assurance (QA) was performed using ArcCHECK diode array, aiming for greater than 95% of points measured to pass a gamma analysis of 3% and 3 mm (Sun Nuclear Corporation, Melbourne, FL, USA). Daily cone beam CT (CBCT) with bone match was used for treatment verification and assessment of bladder filling. In cases of non‐compliance with bladder filling requirements, daily ultrasound scans were used prior to treatment to minimise imaging dose.

## 3 Results

Forty‐three patients were included. Baseline patient and tumour characteristics are listed in Table 1. The median age of patients was 62 (range 37–84); 53.5% had Stage III disease, and 46.5% had clinically involved inguinal or pelvic nodes.

**TABLE 1:** Baseline patient and tumour characteristics.

Patient characteristics	No. of patients	%
Age (years)		
Range	37–84	
Median	62	
Gender		
Male	11	25.6
Female	32	74.4
T stage^a^		
T1	6	13.9
T2	26	60.5
T3	7	16.3
T4	4	9.3
N stage^a^		
N0	23	53.5
N1a	17	39.5
N1b	1	2.3
N1c	2	4.6
Stage^a^		
I	4	9.3
IIA	14	32.6
IIB	2	4.6
IIIA	13	30.2
IIIB	3	7.0
IIIC	7	16.3
Target Volume	Median (cc)	Range (cc)
HR‐PTV/PTV2 (54Gy)	826.8	1722.3
LR‐PTV/PTV1 (45Gy)	2905.2	2565.8

^a^ Staged according to American Joint Cancer Committee Edition 8th (AJCC 8th).

### 3.1 Target Volume

Table 2 details the target volume coverage outcomes evaluated against MNCCI, ANROTAT and RTOG guidelines (eviQ omits recommendations for target volume coverage). Although our local protocol did not mandate optimising specifically to the ANROTAT constraint D98 ≥ 95%–90% of total dose (TD), a median of 93.8% TD and 92.6% TD were achieved for the HR‐PTV and LR‐PTV respectively. The MNCCI constraint D95 ≥ 95%–90% TD and RTOG constraint D95 ≥ 90% TD were achieved for HR‐PTV and PTV‐LR with a median of 96% TD and 95.2% TD respectively. MNCCI protocol also did not mandate optimising to the RTOG constraints D90 of HR‐PTV ≥ 100% of TD or D85 of LR‐PTV ≥ 100% of TD to minimise dose inflation. Thus, these constraints were excluded from this study. Moreover, RTOG and ANROTAT suggested that D2 of both PTVs receive < 115% of TD, without specifying a major violation. This constraint was achieved in 100% of the plans with a median of 105.6% of TD for HR‐PTV and 125.3% of TD for LR‐PTV. However, MNCCI protocol mandated D2 to receive < 110%–115% of TD for the LR‐PTV evaluation structure (eval). The LR‐PTV eval was created by subtracting the HR‐PTV from the LR‐PTV. The D2 constraint for the LR‐PTV eval failed in 67.4% of the plans, with a median of 116.2%.

**TABLE 2:** Target volume coverage achieved in our cohort, according to planning aims from each guideline.

Target volumes				
Guidelines	Planning aims		Results from our cohort	
	Percentage of the PTV volume (%)	Planning aim: to receive a minimum of these doses as a percentage of TD^a^ (%)	Median dose received, as a percentage of TD (Range)	Fail % (Major Violation)
PTV2 (54Gy)				
ANROTAT	D98%	≥ 95% (90% for minor violation)	93.8% (83.5–97.2)	7%
RTOG		≥ 80%^b^		0%
MNCCI		≥ 85% (80% for minor violation)		
RTOG	D95%	≥ 90%^c^	96% (92.9–98.5)	0%
MNCCI		≥ 95% (90% for minor violation)		
RTOG & ANROTAT	D2%	< 115%^d^	105.6% (101.7–109.5)	0%
MNCCI		< 110% (115% for minor violation)		
PTV1 (45Gy)				
ANROTAT	D98%	≥ 95% (90% for minor violation)	92.6% (86.5–97.6)	23%
RTOG		≥ 80%^b^		0%
MNCCI		≥ 85% (80% for minor violation)		
RTOG	D95%	≥ 90%^c^	95.2% (91.2–100.1)	0%
MNCCI		≥ 95% (90% for minor violation)		
RTOG & ANROTAT	D2%	< 115%^e^	125.3% (120.9–129.9)	0%
PTV1 (45Gy) Eval^f^				
MNCCI	D2%	< 110% (115% for minor violation)	116.2% (107.3–123.5)	67.4%

*Note:* RTOG recommends D90% of PTV2 (54Gy) to receive 100% TD and D85% of PTV1 (45Gy) to receive 100% TD. Both constraints have been excluded from this study as they are not implemented in our departmental protocol. This is to avoid overheating the plan.

^a^ TD is an abbreviation for ‘Total Dose’ or ‘Prescription dose’.

^b^ D95% of the PTVs receive ≥ 80% of TD is a minor violation for D98% of PTV to receive ≥ 80% of TD. It is not included in the table as the ideal constraint has been achieved 100% of the time.

^c^ D90% of the PTVs receive ≥ 90% of TD is a minor violation for D95% of PTV to receive ≥ 90% of TD. It is not included in the table as the ideal constraint has been achieved 100% of the time.

^d^ The minor violation for this constraint is any dose > 115% of TD. It is not included in the table as the ideal constraint has been achieved 100% of the time.

^e^ Minor violation of this constraint is any dose > 115% of TD. Locally, D2% of PTV1 (45Gy) is assessed visually in addition to the PTV1 (45Gy) eval to take into account the dose fall off required.

^f^ PTV 1 (45Gy) Eval is the low dose PTV (45Gy) minus the high dose PTV2 (54Gy).

### 3.2 OARs

Tables 3, 4, 5 and Appendices S3–S5 detail the results of dose received to OARs evaluated against recommended dose constraints in Australian and international guidelines and compared to the dose received to OARs in the ANROTAT study. ANROTAT and RTOG specified major and minor violations for target volume coverage, the bowel and femoral heads. However, for the remaining OARs (bladder, iliac crest and external genitalia), the RTOG, ANROTAT and eviQ planning aims were not strict constraints and did not stipulate any major violations but were provided only for plan optimisation.

**TABLE 3:** Dose to gastrointestinal organs at risk according to each guideline.

Gastrointestinal (GI) organs at risk					
Small bowel^a^					Bowel^a^
Guidelines	Planning aims		Results from our cohort		Brown et al. [8]
	Planning aim: volume receiving dose (Gy)	Planning aim: absolute volume receiving dose (cc)	Median (cc) (Range)	Fail % (Major Violation)	Fail % (Major Violation)
MNCCI, RTOG & eviQ	V30	< 200 cc^b^	163.7 cc (10.4–376.9)	40%	N/A
ANROTAT		< 350 cc (450 cc for minor violation)		0%	30%
MNCCI & RTOG	V35	< 150 cc (300 cc for minor violation)	113.3 cc (6.9–298.8)	0%	N/A
eviQ		< 150 cc		35%	
MNCCI, ANROTAT & RTOG	V45	< 20 cc (30 cc for minor violation)	11.5 cc (0–183.2)	9%	73.3%
eviQ		< 20 cc		21%	N/A
MNCCI, ANROTAT, RTOG & eviQ	V50	< 0cc^b^, ^c^	0 cc (0–108.4)	30%	50%

Large bowel^a^, ^d^					Bowel
MNCCI, RTOG & eviQ	V30	< 200 cc	118 cc (15.6–325.8)	14%	N/A
ANROTAT		< 350 cc (450 cc for minor violation)		0%	N/A
MNCCI	V35	< 150 cc (300 cc for minor violation)	80.1 cc (14.5–259.9)	0%	N/A
RTOG & eviQ		< 150 cc		12%	N/A
MNCCI & ANROTAT	V45	< 20 cc (30 cc for minor violation)	15.4 cc (0–130.2)	23%	N/A
RTOG & eviQ		< 20 cc		37%	N/A

^a^ ANROTAT does not clearly specify whether Bowel refers to small bowel alone, large bowel alone or a bowel combination. However, it is defined as mostly small bowel with some large bowel but does not encompass rectosigmoid junction.

^b^ V30Gy < 200 cc and V50Gy < 0 cc do not have a major violation specification and are considered minor violations if the V35Gy < 300 cc and V45Gy < 30 cc are achieved.

^c^ This constraint is defined as a maximum dose < 50Gy on MNCCI, RTOG and EviQ, which can be alternatively notated as V50Gy < 0 cc.

^d^ The large bowel does not have major violation specifications in RTOG and eviQ.

**TABLE 4:** Dose to skeletal organs at risk according to each guideline.

Skeletal organs at risk					
Guidelines	Planning aims		Results from our cohort		Brown et al. [8]
	Planning aim: volume receiving dose (Gy)	Planning aim: as percentage of total volume (%)	Median (Range) (%)	Fail % (Major Violation)	Fail % (Major Violation)
Left Femoral Head					
MNCCI, RTOG and eviQ	V30	< 50%^a^	25.9% (14.3–60.1)	7%	N/A
ANROTAT, RTOG and eviQ	V40	< 35%	5.5% (0.1–17.3)	0%	13.8%
MNCCI		< 35% (45% for minor violation)			N/A
ANROTAT, RTOG, eviQ and MNCCI	V44	< 5% (10% for minor violation)	0.6% (0–7.7)		24.1%
Right Femoral Head					
MNCCI, RTOG & eviQ	V30	< 50%^a^	22.1% (10.2–43.6)	0%	N/A
ANROTAT, RTOG and eviQ	V40	< 35%	6.2% (0.2–16.24)		6.7%
MNCCI		< 35% (45% minor violation)			N/A
ANROTAT, RTOG, eviQ and MNCCI	V44	< 5% (10% minor violation)	0.7% (0–7.04)		26.7%
Iliac Crests^b^					Bone Marrow
ANROTAT, RTOG, eviQ and MNCCI	V30	< 50%	44.96% (31.8–61.7)	19%	23.3%
ANROTAT, RTOG and eviQ	V40	< 35%	22.9% (12.4–35.4)	2%	10%
MNCCI		< 35% (45% for minor violation)		0%	N/A
ANROTAT, RTOG and eviQ	V50	< 5%	0% (0–5.23)	5%	6.7%
MNCCI		< 5% (15% for minor violation)		0%	N/A

^a^ For the femoral heads, V30Gy < 50% does not have a major violation specification. Locally, it is considered a minor violation if the V40Gy < 45% and V44Gy < 10% are achieved. RTOG and ANROTAT specify a major violation if V44Gy < 10% is not achieved.

^b^ RTOG, ANROTAT, eviQ do not have any major violation specifications for the iliac crests. MNCCI does not have a major violation specification for V30Gy < 50% and considers this violation a minor if the V40Gy < 45% and V50Gy < 15% are achieved.

**TABLE 5:** Dose to genitourinary organs at risk according to each guideline.

Genitourinary organs at risk					
Guidelines	Planning aims		Results from our cohort		Brown et al. [8]
	Planning aim: volume receiving dose (Gy)	Planning aim: as percentage of total volume (%)	Median (Range) (%)	Fail % (Major Violation)	Fail % (Major Violation)
External Genitalia^a^					
ANROTAT, RTOG and eviQ	V20	< 50%	49.7% (0–100)	49%	80%
MNCCI		< 50% (70% for minor violation)		23%	N/A
ANROTAT, RTOG and eviQ	V30	< 35%	15.7% (0–99.1)	28%	50%
MNCCI		< 35% (50% for minor violation)		19%	N/A
ANROTAT, RTOG and eviQ	V40	< 5%	4.3% (0–84)	49%	46.7%
MNCCI		< 5% (15% for minor violation)		26%	N/A
Bladder^a^					
ANROTAT, RTOG and eviQ	V35	< 50%	51.8% (38.3–78.3)	56%	73.3%
MNCCI		< 50% (60% for minor violation)		7%	N/A
ANROTAT, RTOG and eviQ	V40	< 35%	33.6% (16.2–68.5)	40%	60%
MNCCI		< 35% (45% for minor violation)		7%	N/A
ANROTAT, RTOG eviQ and	V50	< 5%	1.8% (0–38.3)	37%	40%
MNCCI		< 5% (15% for minor violation)		9%	N/A

^a^ RTOG, ANROTAT and eviQ do not have minor or major violation specifications. MNCCI does not have strict major violation specifications for GU OARs.

#### 3.2.1 Gastrointestinal OAR Constraints

For the small bowel, failure to meet V30Gy < 200 cc (MNCCI, RTOG, eviQ) and V50Gy < 0 cc (MNCCI, ANROTAT, RTOG, eviQ) occurred in 40% and 30% of the plans respectively. Failure to meet the eviQ constraints V35Gy < 150 cc and V45Gy < 20 cc occurred in 35% and 21%, respectively. However, this was considered a minor violation by RTOG and MNCCI if the V35Gy < 150–300 cc and V45Gy < 20–30 cc were achieved. Thus, a major violation only occurred in 9% of plans for V45Gy < 20–30 cc (MNCCI, ANROTAT, RTOG).

For the large bowel, failure to meet V30Gy < 200 cc (MNCCI, RTOG, eviQ), V35Gy < 150 cc (RTOG, eviQ) and V45Gy < 20 cc (RTOG, eviQ) occurred in 14%, 12% and 37% respectively. However, in our departmental protocol, failure to meet V30Gy < 200 cc was considered a minor violation provided that V35Gy < 150–300 cc and V45Gy < 20–30 cc were achieved. There were no major violations for V35Gy < 150–300 cc and major violations occurred only in 23% of the plans for V45Gy < 20–30 cc (MNCCI, ANROTAT).

#### 3.2.2 Skeletal OAR Constraints

For the femoral heads failure to meet V30Gy < 50% occurred in 7% of the plans for the left femoral head (MNCCI, RTOG, eviQ). However, this was locally considered a minor violation as the remaining constraints were achieved. The remaining constraints for the left and right femoral heads achieved 100% compliance without any major violations. ANROTAT and RTOG major violation specification was only for V44Gy < 10%, which was met in 100% of the plans. Thus, no major violations were observed in the femoral head constraints. ANROTAT, eviQ and RTOG did not have any major violation specifications for the iliac crests. However, failure to meet the recommended constraints occurred in 19% for V30Gy < 50% (ANROTAT, RTOG, eviQ, MNCCI), 2% for V40Gy < 35% (ANROTAT, RTOG, eviQ) and 5% for V50Gy < 5% (ANROTAT, RTOG, eviQ). No major violation occurred for the MNCCI constraints V40Gy < 35%–45% and V50Gy < 5%–15%.

#### 3.2.3 Genitourinary (GU) OAR Constraints

ANROTAT, RTOG and eviQ did not have minor or major violation specifications for GU OARs. Failure to meet the external genitalia constraints occurred in 49% of plans for V20Gy < 50%, 28% for V30Gy < 35% and 49% for V40Gy < 5% (RTOG, ANROTAT, eviQ). Major violations only occurred against MNCCI constraints, in 23% of plans for V20Gy < 50%–70%, 19% for V30Gy < 35%–50% and 26% for V40Gy < 5%–15%. For the bladder, 56% of plans failed to achieve the V35Gy < 50% constraint specified by ANROTAT, RTOG and eviQ but only 7% failed the MNCCI V35Gy < 50%–60% constraint. Similarly, 40% of plans failed the V40Gy < 35% constraint for ANROTAT, RTOG and eviQ but only 7% failed the MNCCI V40Gy < 35%–45% constraint. 37% of plans failed the V50Gy < 5% constraint for ANROTAT, RTOG and eviQ but only 9% failed the MNCCI V50Gy < 5%–15% constraint.

## 4 Discussion

There are few reports in the literature regarding whether the recommended planning aims are achievable. We present the dosimetric outcomes of the largest cohort to date of patients treated exclusively with the VMAT technique for curative intent definitive radiation therapy to anal SCC compared against Australian and international planning guidelines to assess the achievability of these recommendations.

Major violations for target volume coverage only occurred against the ANROTAT aims (see Table 2). This is because MNCCI protocol stipulates a lower aim of D98 ≥ 85%. The RTOG and MNCCI aims were fully met. Nevertheless, the clinical significance of achieving the ANROTAT coverage aims (D98 ≥ 90%) is unknown. As published in a separate study for the same patient cohort utilising the MNCCI dose objectives, an excellent 5‐year locoregional control of 90.3% was achieved, with only 4 local failures in total (9.3%) and no regional nodal failures [9]. For comparison, the largest Australian IMRT series [11], the prospective RTOG 0529 trial [1], a Tomotherapy series [12] and a sizable VMAT series [13] reported 5‐year locoregional control rates ranging 80.5%–84%. Therefore, despite not achieving the ANROTAT target volume aims, excellent locoregional control was achieved.

Regarding hotspots, all D2 constraints for HR‐PTV were achieved (see Table 2). Minor violations for the D2 of the LR‐PTV were due to the auto‐contouring margin process used by the RO whereby the LR‐PTV contour overlaps with the HR‐PTV. To ensure compliance with this constraint, the relative hot doses of the LR‐PTV were visually assessed to ensure that there were no large peripheral hotspots. The D2 of the LR‐PTV was also assessed using the LR‐PTV eval to minimise the impact of structure overlap. Overall, target volume coverage and hotspot aims were mostly achieved in our cohort with the aid of a highly conformal VMAT technique.

For gastrointestinal OARs (see Table 3), the median volume received for each constraint was in keeping with, or lower than available reports which have employed an exclusive VMAT technique. Our median small bowel V30Gy was 163.7 cc, as compared to 149 cc [14], 314 cc [3], 229 cc [4] and in the only other Australian study, 217 cc [15]. There are limited reports of V35Gy in the literature. We achieved 113.3 cc, similar to the 108 cc reported by Franco et al. [14]. Our V45Gy was 11.5 cc, which is lower than available reports of 15 cc [14], 31.5 cc [3] and 138 cc [4].

A secondary analysis of the RTOG 0529 trial by Olsen et al. found a statistically significant increase in the risk of Grade 2+ GI toxicity for small bowel loops V30Gy > 155 cc and V35Gy > 41 cc [16]. Lukovic et al. also estimated Grade 2+ GI toxicity to occur in 30% of patients with small bowel loops V35Gy < 60 cc, 40% for V35Gy < 125 cc and 50% for V35Gy < 185 cc [17]. Acute GI toxicity appears to increase in a linear relationship to small bowel V30Gy, without a sudden rise in toxicity rates at any dose threshold [17, 18]. Therefore, while it is ideal to maximally spare the small bowel, this is not always possible. The RO may accept a trade‐off to maximise target volume coverage at the expense of worse small bowel dosimetry. Furthermore, unlike acute toxicity, late GI toxicity does not correlate with the lower dose‐volume metrics (V5 to V45), but instead with V60Gy [16, 18] which was not exceeded in any of our plans. Thus, despite the small bowel being difficult to spare even with highly conformal techniques, failure to meet stringent small bowel dose metrics does not appear to cause adverse long‐term impacts for the patient.

Failure to meet the small bowel dose constraints may be attributed to several factors. The Australian and international guidelines do not consider the overlap between the PTVs and GI OARs. For example, AGITG recommended cropping the small bowel out of the clinical target volume (CTV), but not the PTV, for assessment of the small bowel [6]. However, PTVs often overlap with nearby small bowel loops, which impacts the achievability of small bowel constraints. Female patients were statistically more likely to fail V30, V45 and V50Gy constraints in the ANROTAT study [8]. Other anatomical factors which may impede sparing of the small bowel include the paucity of visceral fat in some patients, and an unfavourable position of the small bowel falling into the rectovesical pouch and rectouterine pouch. Challenges in comparing small bowel dose constraints against other reported studies include the limited number of participants in most studies, heterogeneity in prescription doses, differences in tumour stage of the participants and the anatomical definition of the small bowel contours.

For the large bowel, the physical volume receiving each dose level was again similar to the only available contemporary report, with our median V30Gy, V35Gy and V45Gy being 118 cc, 80.1 cc and 15.4 cc, versus 123 cc, 78 cc and 13 cc respectively by Franco et al. [14]. Thus, despite the presence of major violations, the median volume receiving each dose level in our patients appears to be in keeping with or more optimal than reports in available literature. The clinical impact of these major violations is also questionable, as only 6.8% of our cohort suffered acute Grade 3 GI toxicity, 0% acute Grade 4–5 and 11.3% suffered Grade 2 late GI toxicity and 0% late Grade 3–5 [9].

The skeletal OAR dose constraints were better achieved in our patient cohort utilising exclusive VMAT compared to the ANROTAT study which employed IMRT [8]. However, further fine tuning may be of benefit to address the constraints that were difficult to achieve and tighten the constraints that were easily achievable.

For genitourinary OARs, we noted a lower rate of major violations than ANROTAT except for V40Gy ≤ 5% of the external genitalia. As highlighted in ANROTAT, the major violations for the external genitalia were associated with treatment of inguinal nodes, as the plans did not sacrifice target volume coverage to reduce the dose to the external genitalia [8]. Moreover, the bladder dose‐toxicity correlation has not been well established in the literature [2]. This is further supported by the low toxicities reported in a recent publication for our patient cohort, where only 11.4% and 2.2% of the patients reported acute Grade 2 and 3 genito‐urinary toxicities respectively. Late Grade 2 toxicities occurred in 6.8% of patients. None of our patients had acute Grade 4 or late Grade 3 or 4 toxicities [9]. Variation in bladder preparation protocols across different radiation oncology sites may impact total bladder volume and bladder dosimetry. Appendix S6 details the volumes of targets and OARs.

This study shows that in a patient cohort treated exclusively with VMAT, target coverage aims were achievable. However, the available OAR constraints were not fully achievable. Further refinement of the guidelines may be of benefit to provide realistic dose objectives and constraints. Additionally, the constraints which were easily achievable in our patient cohort would benefit from tightening of the planning objectives.

The strengths of this study include MDT review for all patients, a uniform radiation planning protocol, use of a single treatment planning system, a single RO delineating all target volume and reviewing OAR contours, exclusive VMAT technique and rigorous planning and QA process. The limitations of this study are that it is a retrospective review of plans from a single institution. The small sample size precludes the statistical power to identify significant dose‐volume parameters which may predict acute and late toxicities. Nevertheless, we validated the achievability of planning aims from Australian and international guidelines and from our departmental protocol. This report may benefit other institutions in either auditing existing planning and dosimetric outcomes or in commissioning new pelvic VMAT protocols. It may also assist any international and national attempts to create modern VMAT guidelines for anal SCC. As noted previously, due to a small sample size, the limited number of events either of local recurrence or toxicities, and the overall excellent dosimetric outcomes achieved in our plans, we did not expect any statistical analysis for dosimetric predictors to yield significant findings, and no statistical analysis was performed for this study. Thus, we suggest that future studies in rare entities such as anal cancer would strongly benefit from multi‐institutional prospective registries in order to maximise statistical power and thus identify meaningful dosimetric predictors of clinical outcomes.

## 5 Recommendations

Based upon the results of our cohort, we recommend an updated set of dose constraints (see Table 6). For the target coverage, any dose lower than the minor violation constitutes a major violation. For the OARs, any dose higher than the minor violation is a major violation. The following section highlights the rationale for our proposed recommendations which differ from RTOG, ANROTAT and eviQ.

**TABLE 6:** Recommended dose constraints.

Structure	Ideal constraint	Minor violation
PTV2 (54Gy)	D98% ≥ 90% TD	D98% ≥ 85% TD
	D95% ≥ 95% TD	D95% ≥ 90% TD
	D2% < 107% TD	D2% < 110% TD
PTV1 (45Gy)	D98% ≥ 90% TD	D98% ≥ 85% TD
	D95% ≥ 95% TD	D95% ≥ 90% TD
	LR‐PTV eval D2% < 120% TD^a^	LR‐PTV eval D2% < 125% TD
Small Bowel loops—CTV	V30Gy < 200 cc	V30Gy < 350 cc
	V35Gy < 150 cc	V35Gy < 300 cc
	V45Gy < 20 cc	V45Gy < 30 cc
	Maximum Dose^b^ < 95% TD of the overlapping higher dose PTV	Maximum Dose < 100% of TD of the overlapping higher dose PTV
	Or if there is no overlap. Maximum Dose^c^ < 90% TD of the closest PTV	Maximum Dose < 95% TD of the closest PTV
Femoral Heads	V30Gy < 40%	V30Gy < 50%
	V40Gy < 20%	V40Gy < 35%
	V44Gy < 5%	V44Gy < 10%
Combined Iliac Crests	V30Gy < 50%	V30Gy < 60%
	V40Gy < 30%	V40Gy < 40%
	V50Gy < 0%	V50Gy < 5%
External Genitalia	V20Gy < 50%	V20Gy < 80%
	V30Gy < 35%	V30Gy < 60%
	V40Gy < 5%	V40Gy < 30%
Bladder	V35Gy < 50%	V35Gy < 60%
	V40Gy < 35%	V40Gy < 45%
	V50Gy < 5%	V50Gy < 15%
Large Bowel Loops—CTV	V30Gy < 150 cc	V30Gy < 300 cc
	V35Gy < 100 cc	V35Gy < 200 cc
	V45Gy < 20 cc	V45Gy < 30 cc

^a^ Visual assessment is required to minimise peripheral hotpots.

^b^ When the small bowel loops – CTV overlaps with the PTV, the PTV coverage takes priority over the small bowel dose constraint. The higher dose PTV determines the maximum dose of the small bowel loops—CTV receives if the overlap is with the high dose PTV and vice versa.

^c^ When there is no overlap with the PTV and there is adequate distance for a dose falloff, the closest PTV determines the ideal maximum dose the small bowel loops‐CTV should receive.

### 5.1 Target Volume

We recommend planning aims in between ANROTAT and RTOG. This is because the median D98 achieved for HR‐PTV far exceeded the RTOG constraints. However, the ANROTAT D98 ≥ 95% aims were too stringent, resulting in 7% major and 63% minor violations. Therefore, we have proposed more realistic target coverage objectives of D98 ≥ 90% and increased the major violation threshold to D98 ≥ 85%. The D95 constraint was also tightened to ≥ 95% for ideal and ≥ 90% for minor violation, as 0% of plans incurred a major violation against D95 ≥ 90%.

Similarly for the LR‐PTV, the ANROTAT constraints were too stringent, whereas the RTOG and departmental constraints were fully met. Therefore, we recommend a middle ground for the LR‐PTV to receive D98 ≥ 90% and the major violation threshold at ≥ 85%. The D95 constraint was also tightened to ≥ 95% for ideal and ≥ 90% for minor violation, as all plans met D95 ≥ 90%.

For hotspots in the HR‐PTV, we have suggested a tighter constraint of D2 < 107%, up to < 110% for minor violations, as 100% of our plans met existing aims. Whereas we deemed existing hotspot constraints for the LR‐PTV unfeasible. In order to meet the D95 ≥ 95% objectives for the HR‐PTV, the 51.3Gy isodose line (95% of 54Gy) must conform to the edge of the HR‐PTV. The 51.3Gy isodose would inevitably spill into the adjacent LR‐PTV eval, which constitutes 114% of 45Gy, the TD of the LR‐PTV. The HR‐PTV and LR‐PTV eval were adjoining structures with a 9Gy difference in coverage aims, deeming D2 constraints contradictory. Therefore, D2 of the LR‐PTV eval should be relaxed to < 120%–125% TD.

### 5.2 OARs

#### 5.2.1 GI OARs

For the small bowel loops, we have previously discussed the possible causes of exceeding the existing V30, V35, V40 and V45Gy constraints. Yet, despite the apparent failure to meet these constraints in a significant portion of our plans, this does not appear to impact long‐term patient outcomes. Therefore, we recommend V30Gy < 200 cc and introducing a minor violation of 350 cc, which is stricter than ANROTAT, but less than RTOG and eviQ. Where the small bowel and PTV overlap, we recommend limiting the maximum point dose to the small bowel to 95% TD and 100% TD for minor violation. In instances without overlap, the maximum point dose should be < 90% TD and 95% for minor violation. For the large bowel, V30Gy < 200 cc received a median of 118 cc with a success rate of 86%. Therefore, we recommend modifying the ideal constraint to V30Gy < 150 cc and adding a minor violation of V30Gy < 300 cc. Similarly, the V35Gy < 150–300 cc constraint was achieved with a median of 80 cc, and 88% of the plans were compliant. We thus recommend a stricter ideal constraint of V35Gy < 100 cc and a minor violation of V35Gy < 200 cc.

#### 5.2.2 Skeletal OARs

The femoral heads achieved low median doses and high compliance rates. Therefore, we recommend tightening both the ideal and minor violation specifications to V30Gy < 40%–50% and V40Gy < 20%–35%. For the Iliac crests, V30Gy < 50% failed in 19% of the plans with a median of 44.96% and a maximum of 61.7%. We thus recommend adding a minor violation specification of V30Gy < 60%. V40Gy < 35% and V50Gy < 5% were achieved in 98 and 95% of the plans. Hence, we recommend a stricter V40Gy < 30%–40% and V50Gy < 0%–5%.

#### 5.2.3 GU OARs

The departmental external genitalia V20, V30 and V40Gy constraints failed in 19%–26% of patients. We thus recommend loosening the minor violation threshold by a further 10%–15%. The bladder constraints from RTOG/ANROTAT were difficult to meet. However, with the addition of minor violation specifications in our local departmental protocol, most plans were able to pass. We recommend loosening the minor violation thresholds to ensure realistic bladder constraints.

## 6 Conclusion

By employing exclusively VMAT at a regional Australian centre, our department was able to achieve high compliance rates with ANROTAT, RTOG, eviQ and departmental constraints. Our OAR doses were lower than those in other VMAT reports in the literature and ANROTAT, which utilised IMRT. This study validates the achievability of the current planning aims and recommends modified objectives based on clinically driven data. Nonetheless, further studies are required to evaluate the clinical implications and utility of these recommended constraints.

## Funding

The authors have nothing to report.

## Ethics Statement

Institutional ethics approval by the North Coast NSW Human Research Ethics Committee (NCNSW 2021/ETH00346).

## Conflicts of Interest

The authors declare no conflicts of interest.

## Supporting information


**Appendix S1:** (A) Target volume coverage achieved in our cohort, according to planning aims from each guideline. (B) D2% for the PTV1 (45Gy) Eval.
**Appendix S2:** An example of an approved plan.
**Appendix S3:** Dose to Gastrointestinal Organs at risk according to each guideline.
**Appendix S4:** Dose to Skeletal Organs at risk according to each guideline.
**Appendix S5:** Dose to Genitourinary Organs at risk according to each guideline.
**Appendix S6:** Targets and OARs Volumes.

## Data Availability

The data supporting this study's findings are available from the corresponding author upon reasonable request.

## References

[1] L. A. Kachnic , K. A. Winter , R. J. Myerson , et al., “Long‐Term Outcomes of NRG Oncology/RTOG 0529: A Phase 2 Evaluation of Dose‐Painted Intensity Modulated Radiation Therapy in Combination saWith 5‐Fluorouracil and Mitomycin‐C for the Reduction of Acute Morbidity in Anal Canal Cancer,” International Journal of Radiation Oncology, Biology, Physics 112, no. 1 (2022): 146–157. 10.1016/j.ijrobp.2021.08.008 PMC8688291 34400269

[2] L. A. Kachnic , K. Winter , R. J. Myerson , et al., “RTOG 0529: A Phase 2 Evaluation of Dose‐Painted Intensity Modulated Radiation Therapy in Combination With 5‐Fluorouracil and Mitomycin‐C for the Reduction of Acute Morbidity in Carcinoma of the Anal Canal,” International Journal of Radiation Oncology, Biology, Physics 86, no. 1 (2013): 27–33. 10.1016/j.ijrobp.2012.09.023 PMC3619011 23154075

[3] S. Vieillot , D. Azria , C. Lemanski , et al., “Plan Comparison of Volumetric‐Modulated Arc Therapy (RapidArc) and Conventional Intensity‐Modulated Radiation Therapy (IMRT) in Anal Canal Cancer,” Radiation Oncology 5 (2010): 92. 10.1186/1748-717X-5-92 PMC2965153 20942958

[4] H. Mok , T. M. Briere , M. K. Martel , et al., “Comparative Analysis of Volumetric Modulated Arc Therapy Versus Intensity Modulated Radiation Therapy for Radiotherapy of Anal Carcinoma,” Practical Radiation Oncology 1, no. 3 (2011): 163–172. 10.1016/j.prro.2011.01.003 24673946

[5] A. Clivio , A. Fogliata , A. Franzetti‐Pellanda , et al., “Volumetric‐Modulated Arc Radiotherapy for Carcinomas of the Anal Canal: A Treatment Planning Comparison With Fixed Field IMRT,” Radiotherapy and Oncology 92, no. 1 (2009): 118–124. 10.1016/j.radonc.2008.12.020 19181409

[6] M. Ng , T. Leong , S. Chander , et al., “Australasian Gastrointestinal Trials Group (AGITG) Contouring Atlas and Planning Guidelines for Intensity‐Modulated Radiotherapy in Anal Cancer,” International Journal of Radiation Oncology, Biology, Physics 83, no. 5 (2012): 1455–1462. 10.1016/j.ijrobp.2011.12.058 22401917

[7] R. J. Myerson , M. C. Garofalo , I. El Naqa , et al., “Elective Clinical Target Volumes for Conformal Therapy in Anorectal Cancer: A Radiation Therapy Oncology Group Consensus Panel Contouring Atlas,” International Journal of Radiation Oncology, Biology, Physics 74, no. 3 (2009): 824–830. 10.1016/j.ijrobp.2008.08.070 PMC2709288 19117696

[8] E. Brown , A. Cray , A. Haworth , et al., “Dose Planning Objectives in Anal Canal Cancer IMRT: The TROG ANROTAT Experience,” Journal of Medical Radiation Sciences 62, no. 2 (2015): 99–107. 10.1002/jmrs.99 PMC4462981 26229674

[9] M. T. Hsieh , T. P. Shakespeare , L. Winkley , D. Goonetilleke , S. Z. L. Yap , and A. R. M. Tahir , “Long‐Term Outcomes of Definitive Radiation With Volumetric Modulated Arc Therapy and Concurrent Chemotherapy for Squamous Cell Carcinoma of the Anus in a Regional Australian Cancer Centre,” Journal of Medical Imaging and Radiation Oncology 68, no. 3 (2024): 325–332. 10.1111/1754-9485.13630 38450897

[10] 1860‐Anal Definitive EBRT Chemoradiation | eviQ . [Cited 2023 Nov 22], https://www.eviq.org.au/radiation‐oncology/colorectal/1860‐anal‐definitive‐ebrt‐chemoradiation.

[11] J. Sia , W. M. Lim , R. A. Agas , et al., “Long‐Term Patterns of Failure and the Value of Blood Prognostic Markers in Anal Cancers Treated With Intensity‐Modulated Radiation Therapy,” Clinical Colorectal Cancer 21, no. 2 (2022): e102–e112. 10.1016/j.clcc.2021.10.005 34799240

[12] B. De Bari , R. Jumeau , H. Bouchaab , et al., “Efficacy and Safety of Helical Tomotherapy With Daily Image Guidance in Anal Canal Cancer Patients,” Acta Oncologica 55, no. 6 (2016): 767–773. 10.3109/0284186X.2015.1120886 27034083

[13] J. Possiel , H. E. Ammon , M. Guhlich , et al., “Volumetric Modulated Arc Therapy Improves Outcomes in Definitive Radiochemotherapy for Anal Cancer Whilst Reducing Acute Toxicities and Increasing Treatment Compliance,” Cancers 13, no. 11 (2021): 2533. 10.3390/cancers13112533 PMC8196749 34064061

[14] P. Franco , F. Arcadipane , R. Ragona , et al., “Volumetric Modulated Arc Therapy (VMAT) in the Combined Modality Treatment of Anal Cancer Patients,” British Journal of Radiology 89, no. 1060 (2016): 20150832. 10.1259/bjr.20150832 PMC4846209 26838953

[15] E. Brown , E. Le Cornu , T. Bui , A. Bernard , T. Mai , and J. Harvey , “Acute Toxicity and Patient‐Reported Outcomes in Anal Canal Cancer: Results of a Pilot Study,” Journal of Medical Radiation Sciences 69, no. 4 (2022): 484–491. 10.1002/jmrs.604 PMC9714504 35751555

[16] J. R. Olsen , J. Moughan , R. Myerson , et al., “Predictors of Radiation Therapy–Related Gastrointestinal Toxicity From Anal Cancer Dose‐Painted Intensity Modulated Radiation Therapy: Secondary Analysis of NRG Oncology RTOG 0529,” International Journal of Radiation Oncology, Biology, Physics 98, no. 2 (2017): 400–408. 10.1016/j.ijrobp.2017.02.005 PMC5639877 28463160

[17] J. Lukovic , A. Hosni , A. Liu , et al., “Evaluation of Dosimetric Predictors of Toxicity After IMRT With Concurrent Chemotherapy for Anal Cancer,” Radiotherapy and Oncology 178 (2022): 109429. 10.1016/j.radonc.2022.11.018 36455685

[18] K. S. Storm , K. L. G. Spindler , G. F. Persson , et al., “Predictors of Acute and Late Diarrhea in the Treatment of Anal Cancer With Concurrent Chemoradiotherapy,” Acta Oncologica 64 (2025): 1217–1226. 10.2340/1651-226X.2025.43975 PMC12449700 40955104

