# Supplementary material for: Dosimetric Outcomes of Definitive Radiation Therapy to Anal Squamous Cell Carcinoma in a Single Institution Using Volumetric Modulated Arc Therapy Evaluated Against Australian and International Treatment Planning Guidelines
Source: J Med Radiat Sci. 2026 Oct 29:10.1002/jmrs.70124. Online ahead of print. doi: 10.1002/jmrs.70124 (PMC13717669; doi:10.1002/jmrs.70124)
Supplement: Supplementary file 1 [file JMRS-9999-0-s001.docx]

***Appendix 1A:* Target volume coverage achieved in our cohort, according to planning aims from each guideline.**

|  | PTV2(54Gy) | | | | | | |
| --- | --- | --- | --- | --- | --- | --- | --- |
|  | D98% | | | D95% | | D2% | |
|  | ≥85% of TD (80% for minor violation) | ≥80% of TD^1^ | ≥95% of TD (90% for minor violation) | ≥95% of TD (90% for minor violation) | ≥90% of TD^2^ | ≤110% of TD (115% for minor violation) | ≤115% of TD^3^ |
|  | MNCCI | RTOG | ANROTAT | MNCCI | RTOG | MNCCI | RTOG & ANROTAT |
| Mean (%) | 93.7% | | | 95.98% | | 105.7% | |
| Standard deviation (%) | 2.5% | | | 1.4% | | 1.5% | |
| Median (%) | 93.7% | | | 96% | | 105.6% | |
| Max (%) | 97.1% | | | 98.5% | | 109.5% | |
| Min (%) | 83.5% | | | 92.9% | | 101.7% | |
| Range (%) | 13.6% | | | 5.5% | | 7.8% | |
| % of plans achieving ideal constraint | 98% | 100% | 30% | 84% | 100% | 100% | |
| % of plans with minor violation | 2% | 0% | 63% | 16% | 0% | 0% | |
| % of plans with major violation | 0% | 0% | 7% | 0% | 0% | N/A | |
| PTV1(45Gy) | | | | | | | |
|  | D98% | | | D95% | | D2% | |
|  | ≥85% of TD (80% for minor violation) | ≥80% of TD^1^ | ≥95% of TD (90% for minor violation) | ≥95% of TD (90% for minor violation) | ≥90% of TD^2^ | ≤110% of TD (115% for minor violation) | ≤115% of TD^3,4^ |
|  | MNCCI | RTOG | ANROTAT | MNCCI | RTOG | MNCCI | RTOG & ANROTAT |
| Mean (%) | 92.3% | | | 95.4% | | 125.5% | |
| Standard deviation (%) | 2.7% | | | 2% | | 1.1% | |
| Median (%) | 92.5% | | | 95.2% | | 125.3% | |
| Max (%) | 97.6% | | | 100.1% | | 129.9% | |
| Min (%) | 86.5% | | | 91.2% | | 120.9% | |
| Range (%) | 11.1% | | | 8.9% | | 9.1% | |
| % of plans achieving ideal constraint | 100% | | 12% | 70% | 100% | 0% | 0% |
| % of plans with minor violation | 0% | | 65% | 30% | 0% | 0% | 100% |
| % of plans with major violation | 0% | | 23% | 0% | 0% | 100% | N/A |

1. D95% of the PTVs to receive ≥ 80% of TD is a minor violation for D98% of PTV to receive ≥ 80% of TD. It is not included in the table as the ideal constraint has been achieved 100% of the time.
2. D90% of the PTVs receive ≥ 90% of TD is a minor violation for D95% of PTV to receive ≥ 90% of TD. It is not included in the table as the ideal constraint has been achieved 100% of the time.
3. The minor violation for this constraint is any dose ≥ 115% of TD. It is not included in the table as the ideal constraint has been achieved 100% of the time.
4. D2% of PTV1(45) is assessed on PTV 1(45Gy) eval and visually in our protocols to minimise the impact of PTV overlap.
5. TD is an abbreviation for “Total Dose” or “Prescription dose”.

***Appendix 1B:* D2% for the PTV1 (45Gy) Eval**

| **PTV1(45Gy) Eval** | |
| --- | --- |
| MNCCI |  |
| D2% | <110% (115% for minor violation)^1^ |
| Mean (%) | 116.4% |
| Standard deviation (%) | 1.5% |
| Median (%) | 116.2% |
| Max (%) | 123.5 |
| Min (%) | 107.3% |
| Range (%) | 16.4% |
| % of plans achieving ideal constraint | 4.6% |
| % of plans with minor violation | 27.9% |
| % of plans with major violation | 67.4% |

1 _This constraint does not account for the necessary distance to achieve an adequate dose fall-off, and it is evaluated alongside a visual review of hotspot locations_

***Appendix 2: An example of an approved plan.***

***Appendix 3:* Dose to Gastrointestinal Organs at risk according to each guideline**

| **Gastrointestinal (GI) Organs at Risk** | | | | | | | |
| --- | --- | --- | --- | --- | --- | --- | --- |
| **Small Bowel** | | | | | | | |
|  | V30Gy | | V35Gy | | V45Gy | | V50Gy |
|  | ≤200cc | ≤350cc (450cc for minor violation) | ≤150cc (300cc for minor violation) | ≤150cc | ≤20cc (30cc for minor violation) | ≤20cc | ≤0cc |
|  | MNCCI, RTOG & eviQ | ANROTAT | MNCCI & RTOG | ANROTAT & eviQ | MNCCI, ANROTAT & RTOG | eviQ | MNCCI, ANROTAT, RTOG & eviQ |
| Mean (cc) | 171.6cc | | 128.7cc | | 18.3cc | | 2.99cc |
| Standard deviation (cc) | 106.8cc | | 86.4cc | | 31.4cc | | 16.6cc |
| Median (cc) | 163.7cc | | 113.3cc | | 11.5cc | | 0cc |
| Max (cc) | 376.9cc | | 298.8cc | | 183.2cc | | 108.4cc |
| Min (cc) | 10.4cc | | 6.9cc | | 0cc | | 0cc |
| Range(cc) | 366.5cc | | 291.9cc | | 183.2cc | | 108.4cc |
| % of plans achieving ideal constraint | 60% | 93% | 65% | 65% | 79% | 79% | 70% |
| % of plans with minor violation | N/A | 7% | 35% | N/A | 12% | N/A | N/A |
| % of plans with failure (or major violation) | 40% | 0% | 0% | 35% | 9% | 21% | 30% |
| **Large Bowel** | | | | | | | |
|  | V30Gy | | V35Gy | | V45Gy | | N/A |
|  | ≤200cc | ≤350cc (450cc for minor violation) | ≤150cc (300cc for minor violation) | ≤150cc | ≤20cc (30cc for minor violation) | ≤20cc | N/A |
|  | MNCCI, RTOG & eviQ | ANROTAT | MNCCI | ANROTAT, RTOG & eviQ | MNCCI & ANROTAT | RTOG & eviQ | N/A |
| Mean (cc) | 125.4cc | | 95.2cc | | 24.7cc | | N/A |
| Standard deviation (cc) | 71.5cc | | 57.8cc | | 25.8cc | | N/A |
| Median (cc) | 118cc | | 80.2cc | | 15.4cc | | N/A |
| Max (cc) | 325.8cc | | 259.9cc | | 130.2cc | | N/A |
| Min (cc) | 15.6cc | | 14.5cc | | 0cc | | N/A |
| Range(cc) | 310.1cc | | 245.4cc | | 130.2cc | | N/A |
| % of plans achieving ideal constraint | 86% | 100% | 88% | 88% | 63% | 63% | N/A |
| % of plans with minor violation | N/A | 0% | 12% | N/A | 14% | N/A | N/A |
| % of plans with failure (or major violation) | 14% | 0% | 0% | 12% | 23% | 37% | N/A |

***Appendix 4:* Dose to Skeletal Organs at risk according to each guideline**

| **Skeletal Organs at Risk** | | | | |
| --- | --- | --- | --- | --- |
| **Left Femoral Head** | | | | |
|  | V30Gy | V40Gy | | V44Gy |
|  | ≤50% | ≤35% (45% for minor violation) | ≤35% | ≤5% (10% for minor violation) |
|  | MNCCI, RTOG & eviQ | MNCCI | ANROTAT, RTOG & eviQ | MNCCI, ANROTAT, RTOG & eviQ |
| Mean (%) | 28.8% | 7% | | 1.7% |
| Standard deviation (%) | 11.4% | 4.9% | | 2.2% |
| Median (%) | 25.9% | 5.5% | | 0.6% |
| Max (%) | 60.1% | 17.3% | | 7.7% |
| Min (%) | 14.7% | 0.1% | | 0% |
| Range (%) | 45.3% | 17.1% | | 7.7% |
| % of plans achieving ideal constraint | 93% | 100% | 100% | 88% |
| % of plans with minor violation | N/A | 0% | N/A | 12% |
| % of plans with failure (or major violation) | 7% | 0% | 0% | 0% |
| **Right Femoral Head** | | | | |
|  | V30Gy | V40Gy | | V44Gy |
|  | ≤50% | ≤35% (+10% for minor violation) | ≤35% | ≤5% (10% for minor violation) |
|  | MNCCI, RTOG & eviQ | MNCCI | ANROTAT, RTOG & eviQ | MNCCI, ANROTAT, RTOG & eviQ |
| Mean (%) | 23.7% | 6.4% | | 1.7% |
| Standard deviation (%) | 7.9% | 4.3% | | 2.1% |
| Median (%) | 22.1% | 6.2% | | 0.7% |
| Max (%) | 43.6% | 16.2% | | 7% |
| Min (%) | 10.2% | 0.2% | | 0% |
| Range (%) | 33.4% | 16% | | 7% |
| % of plans achieving ideal constraint | 100% | 100% | 100% | 88% |
| % of plans with minor violation | 0% | 0% | N/A | 12% |
| % of plans with failure (or major violation) | 0% | 0% | 0% | 0% |

| **Iliac Crests** | | | | | |
| --- | --- | --- | --- | --- | --- |
|  | V30Gy | V40Gy | | V50Gy | |
|  | ≤50% | ≤35% (45% for minor violation) | ≤35% | ≤5% (15% for minor violation) | ≤5% |
|  | MNCCI, ANROTAT, RTOG & eviQ | MNCCI | ANROTAT, RTOG & eviQ | MNCCI | ANROTAT, RTOG & eviQ |
| Mean (%) | 44.8% | 23.1% | | 0.5% | |
| Standard deviation (%) | 7.6% | 5.8% | | 1.3% | |
| Median (%) | 44.96% | 22.9% | | 0% | |
| Max (%) | 61.7% | 35.4% | | 5.2% | |
| Min (%) | 31.8% | 12.4% | | 0% | |
| Range (%) | 29.9% | 22.99% | | 5.2% | |
| % of plans achieving ideal constraint | 81% | 98% | 98% | 95% | 95% |
| % of plans with minor violation | N/A | 2% | N/A | 5% | N/A |
| % of plans with failure (or major violation) | 19% | 0% | 2% | 0% | 5% |

***Appendix 5:* Dose to Genitourinary Organs at risk according to each guideline**

| **Genitourinary Organs at Risk** | | | | | | |
| --- | --- | --- | --- | --- | --- | --- |
| **External Genitalia** | | | | | | |
|  | V20Gy | | V30Gy | | V40Gy | |
|  | ≤50% (70% for minor violation) | ≤50% | ≤35% (50% for minor violation) | ≤35% | ≤5% (15% for minor violation) | ≤5% |
|  | MNCCI | ANROTAT, RTOG & eviQ | MNCCI | ANROTAT, RTOG & eviQ | MNCCI | ANROTAT, RTOG & eviQ |
| Mean (%) | 55.1% | | 27.6% | | 15.99% | |
| Standard deviation (%) | 27.9% | | 29.7% | | 24.5% | |
| Median (%) | 49.7% | | 15.7% | | 4.3% | |
| Max (%) | 100% | | 99.1% | | 84% | |
| Min (%) | 0% | | 0% | | 0% | |
| Range (%) | 100% | | 99.1% | | 84% | |
| % of plans achieving ideal constraint | 51% | 51% | 72% | 72% | 51% | 51% |
| % of plans with minor violation | 26% | N/A | 9% | N/A | 23% | N/A |
| % of plans with failure (or major violation) | 23% | 49% | 19% | 28% | 26% | 49% |
| **Bladder** | | | | | | |
|  | V35Gy | | V40Gy | | V50Gy | |
|  | ≤50% (60% for minor violation) | ≤50% | ≤35% (45% for minor violation) | ≤35% | ≤5% (15% for minor violation) | ≤5% |
|  | MNCCI | ANROTAT, RTOG & eviQ | MNCCI | ANROTAT, RTOG & eviQ | MNCCI | ANROTAT, RTOG & eviQ |
| Mean (%) | 51.6% | | 34.7% | | 5.5% | |
| Standard deviation (%) | 7.9% | | 9.9% | | 8.2% | |
| Median (%) | 51.8% | | 33.6% | | 1.8% | |
| Max (%) | 78.3% | | 68.5% | | 38.3% | |
| Min (%) | 38.3% | | 16.2% | | 0% | |
| Range (%) | 40% | | 52.3% | | 38.3% | |
| % of plans achieving ideal constraint | 44% | 44% | 60% | 60% | 63% | 63% |
| % of plans with minor violation | 49% | N/A | 33% | N/A | 28% | N/A |
| % of plans with failure (or major violation) | 7% | 56% | 7% | 40% | 9% | 37% |

***Appendix 6:* Targets and OARs Volumes**

| **Targets and OARs Volumes in CC** | | | | | |
| --- | --- | --- | --- | --- | --- |
|  | **Targets** | | | **GI OARs** | |
|  | **PTV2(54Gy)** | **PTV1(45Gy)** | **PTV1(45Gy) Eval** | **Small Bowel** | **Large Bowel** |
| **Mean (cc)** | 906.0 | 2859.4 | 1913.7 | 410.6 | 275.1 |
| **Standard deviation (cc)** | 360.3 | 587.2 | 576.0 | 220.2 | 148.8 |
| **Median (cc)** | 826.8 | 2905.2 | 1872.6 | 423.1 | 220.0 |
| **Min (cc)** | 257.6 | 1786.2 | 892.3 | 37.1 | 69.5 |
| **Max (cc)** | 1979.9 | 4352.0 | 3234.4 | 870.0 | 694.2 |
| **Range (cc)** | 1722.3 | 2565.8 | 2342.1 | 832.9 | 624.7 |
|  | **Skeletal OARs** | | | **Genitourinary OARs** | |
|  | **L Femoral Head** | **R Femoral Head** | **Iliac Crests** | **External Genitalia** | **Bladder** |
| **Mean (cc)** | 148.7 | 151.0 | 456.5 | 154.6 | 309.5 |
| **Standard deviation (cc)** | 32.8 | 32.1 | 123.9 | 144.0 | 113.6 |
| **Median (cc)** | 142.3 | 141.4 | 427.0 | 104.0 | 300.5 |
| **Min (cc)** | 96.9 | 101.5 | 270.0 | 16.7 | 83.0 |
| **Max (cc)** | 253.7 | 254.1 | 1027.1 | 668.4 | 533.3 |
| **Range (cc)** | 156.8 | 152.6 | 757.1 | 651.7 | 450.3 |
